# Experiences of healthcare professionals in a breastfeeding training program

**DOI:** 10.1186/s13006-025-00760-2

**Published:** 2025-08-12

**Authors:** Karin Cato, Eva-Lotta Funkquist, Paola Oras

**Affiliations:** Department of Women’s and Children’s Health, Uppsala University, Dag Hammarskjölds väg 14 B, Uppsala, 752 37 Sweden

**Keywords:** Breastfeeding, Health care professionals, Training program

## Abstract

**Objective:**

This study aimed to elucidate healthcare professionals’ (HCPs) evaluations of a breastfeeding training program that incorporated diverse professions along the care continuum.

**Methods:**

The breastfeeding training program was conducted over a full day during 2018–2019. To enable as many HCPs as possible to participate, the program was offered om twelve different dates. Approximately 25 HCPs took part on each occasion. Both qualitative and quantitative data was gathered on each occasion. The cohort comprised 238 HCPs, including midwives, registered nurses, specialist registered nurses, assistant nurses, physicians, and psychologists, all actively engaged in clinical practice at delivery/maternity wards or child healthcare centers. HCPs completed questionnaires featuring both closed and open-ended queries at the commencement and conclusion of the training program. Additionally, participants collaborated in small groups to propose improvements within the care continuum.

**Results:**

Following the training program, HCPs reported a perceived increase in their interest in breastfeeding and noted the acquisition of novel tools for breastfeeding support. Noteworthy aspects of the training program, as identified by participants, included group discussions, the structure of the training session, inspiration for breastfeeding support, newfound knowledge regarding breastfeeding, and the utility of provided parental materials.

**Conclusion:**

The breastfeeding training program was beneficial across various HCP roles. The training program served to augment participants’ interest in breastfeeding and equipped them with resources to bolster ongoing breastfeeding support efforts. Facilitating HCP attendance at such training sessions and fostering commitment to breastfeeding promotion emerge as crucial imperatives.

**Trial registration:**

Detailed information about the program and its implementation is available in the trial registered in the ISRCTN Registry: 10.1186/ISRCTN91972905.

**Supplementary Information:**

The online version contains supplementary material available at 10.1186/s13006-025-00760-2.

## Introduction

Breastfeeding is widely documented as the optimal source of nutrition for infants, providing many health benefits for both the child and the mother and recommended exclusively for the first six months [1–3]. However, many women face challenges with breastfeeding, and the support they receive from health care professionals (HCPs) plays a crucial role in their breastfeeding journey [4]. Two systematic reviews have recently evaluated studies on breastfeeding skills education for HCPs [5, 6]. The researchers conclude that there still is a need for research evidence to optimize the design and delivery of educations, but in most countries a limited attention is given to breastfeeding education within education systems [7], which we also recognize from the Swedish context. The training in breastfeeding among HCPs is often not a prioritized area and one of the problems are that the composition of HCPs involved in breastfeeding support varies, with different HCPs holding different levels of expertise and experience in this area [6].

The provision of comprehensive perinatal care is central to promoting maternal and infant health and well-being. In Sweden, mothers to be meet a well-established healthcare system comprising various institutions tailored to meet their diverse needs throughout the perinatal journey. Antenatal care clinics serve as the primary point of contact for pregnant women and breastfeeding education is part of their program with personalized care plans. The maternity hospitals provide obstetric and neonatal care to individuals during childbirth and breastfeeding support is a part of their mission [8].

Support from HCPs with good knowledge and positive attitudes to breastfeeding is associated with better breastfeeding outcomes and breastfeeding training programs can have impact on both knowledge and attitudes. Recommendations in the form of clinical guidelines are established to optimize healthcare. The Baby-Friendly Hospital Initiative (BFHI) serves as an example of guidelines concerning breastfeeding [1]. However, the mere formulation of guidelines does not ensure adherence. There are numerous reasons for this, including time constraints, low expectations regarding outcomes, or lack of knowledge. Bridging the gap between research and clinical practice necessitates education [9]. For such education to be effective, it must be perceived as meaningful by the staff and feasible by the employer. The educational program described in this study is developed using Intervention Mapping, a method for implementing complex interventions [10]. An integral part of the intervention is assessing its feasibility of the HCPs who are intended to be part of it.

### Aim

This study aimed to elucidate healthcare professionals’ (HCPs) evaluation of a breastfeeding training program that incorporated diverse professions along the care continuum.

## Methods

Both a quantitative and qualitative approach was used to evaluate a breastfeeding training program for HCPs as part of a larger implementation project on improved breastfeeding support.

### Design

The present study is based on the collected qualitative and quantitative data about the HCPs experiences and perspectives of breastfeeding support. Individual questionnaires with Likert scales and open-ended questions were answered by the participants before and after the training program. In small groups of different professions, the HCPs took notes on suggestions of improvement regarding their work with breastfeeding support.

### Setting

The breastfeeding training program was offered to health care professionals working in two Swedish counties with approximately 450 000 inhabitants. Both counties have hospitals with obstetric health care were nearly 4500 and 500 infants are born yearly respectively. During pregnancy, the expectant woman and her partner visit a midwife at the antenatal clinic 6–10 times. The median hospital stay for families giving birth is one day at the larger hospital and two days at the smaller hospital. After discharge, families from the larger hospital receive follow-up care at home from midwives stationed at the hospital. Rooming-in is practiced in the postnatal wards, where families may interact with physicians, midwives, registered nurses, specialist registered nurses, assistant nurses, and psychologists. The birthing woman is offered follow-up appointments with a midwife at the antenatal clinic, and the child is enrolled in the child care center, where registered nurses or specialist nurses provide care.

### Sample

Midwives, registered nurses, specialist registered nurses, assistant nurses, physicians and psychologists, working clinically at prenatal clinics, delivery/postnatal ward or child care centers, attended the training program. Of 268 participating HCPs, a total of 238 (89%) HCPs choose to participate in the study and filled in questionnaires at the beginning and at the end of the training program.

### Description of the training program

The breastfeeding training program was part of a larger breastfeeding project, which had the BFHIs Ten Steps as its foundation, and was developed with the assistance of Intervention Mapping [11]. The program included material for both HCPs as well as for parents of both preterm and full-term infants. Results for studies of the training program with HCPs working at the NICU, are to be found elsewhere [12]. The part of the training program targeting HCPs included eight web lectures with a self-test at the end of the lecture for those who wanted to test their knowledge after watching. The HCPs were able to watch the lectures during work hours. After watching the web lectures, the HCPs attended an 8-h training program, starting with a recapitulation of the web lectures and a discussion of its content. Thereafter, the HCPs were randomly placed in smaller groups of five HCPs in each group with the following questions/statements to discuss; (a) Personal experience of breastfeeding, (b) Working with breastfeeding in your professional role, and (c) What are your suggestions for improvements in breastfeeding support in your clinical work/setting? During the discussion, the groups wrote down their proposed improvements. These notes were collected by the researchers in order to later analyze which aspects the HCPs considered important for improving work related to breastfeeding. Later on, the smaller groups came together as a whole group again, and reflections on personal experiences, working with breastfeeding support as well as implementation of proposed improvements where discussed. For most of the training program, the course focused on different breastfeeding scenarios/cases. Again, the HCPs were divided into smaller groups were given three scenarios per group, out of the total 17 breastfeeding scenarios (Appendix 1), to discuss in depth. Thereafter, all 17 scenarios were discussed in the large group. The course leader moderated the discussions in the large group and highlighted important aspects of each scenarios if not already mentioned by the HCPs.

### Data collection

The training program (where the discussion day were given at 12 different dates) were conducted in 2018 to 2019 and were headed by researchers within the study project. Oral and written information was given to the HCPs, explaining that their enrollment in the study was voluntary and that they had the right to withdraw their participation at any time. At the start of the training program, the participants were asked to answer a questionnaire about background characteristics and rate their interest in working with breastfeeding support anonymously as well as their thoughts on how important their professional role was in giving breastfeeding support. Background characteristics included in the questionnaire were; age, sex, education, profession and years in the profession. The questions on interest in breastfeeding and importance of their role were answered on a scale ranging from 1 = *Not correct at all* to 10 = *Absolutely right*, and 1 = *Not important at all* to 10 = *Very important*. At the very end of the training program, the participating HCPs answered another questionnaire if their interest in breastfeeding had increased during the training and if they had received new tools to use when giving breastfeeding support. Both questions were answered on a scale ranging from 1 = *Not correct at all* to 10 = *Absolutely right*. The questionnaire also included open free text questions were the participants could share what was good about the training program and if something about the training program could be improved.

### Data analysis

The characteristics of the participating HCPs are presented by descriptive statistics. When analyzing median differences in how the HCPs answered the 10-point scales, the Kruskal-Wallis test was used, while Spearman’s rho was used for correlation analyses. The level of statistical significance was set a p-value of < 0.05. The suggestions for improvements regarding breastfeeding support at work, that the participating HCPs wrote down on worksheets, were analyzed using qualitative content analysis, as described by Graneheim & Lundman [13], where initial codes and categories were identified by the first author. The open-ended questions from the questionnaire were analyzed by summative content analysis. How did the HCPs experience the breastfeeding training program was evaluated through two open-ended questions, namely: “What was particularly good about the training program?” (157 answers) and “How can we improve the training program?” (78 answers). Six categories were finally confirmed as representing the positive experiences of the training program according to the participants and five categories represented their suggestions for improvement of the training program.

All the qualitative data was read by all authors and codes and categories were discussed and finally agreed upon. Throughout the process, the research group kept awareness of their pre-understanding of the subject as researchers and midwives.

## Results

Background characteristics for the participating HCPs are to be found in Table 1. All HCPs, except four were female. Participating HCPs consisted of midwives, registered nurses, specialist registered nurses, assistant nurses, physicians and psychologists. The mean age of the HCPs was 45 and mean years in their profession was 14.

**Table 1:** Background characteristics for the participants (*n* = 237*) of the training program

Participants	Value
Mean age in years (range)	45 (24–67)
Mean years in profession (range) Midwife, *n* (%)	14 (0.5–45) 142 (60)
Registered nurse, *n* (%)	7 (3)
Specialist registered nurse, *n* (%)	14 (6)
Assistant nurse, *n* (%)	60 (25)
Physician, *n* (%) Psychologist, *n* (%)	12 (5) 2 (1)

*Background characteristic missing for one participant

When beginning the training program, the HCPs reported their interest in breastfeeding as a median (range) of 8 (2–10) points on the 10-point scale. The HCPs rated their importance of their role in providing improved breastfeeding support in the care chain as a median (range) of 7 (3–10) points. The HCPs’ age correlated with how they rated their importance of their role in providing breastfeeding support (r_s_= 0,158, p = 0,016). The older age, the more important they rated their role. The Kruskal-Wallis test showed that there was a significant difference between the midwives’, registered nurses’, specialist registered nurses’, assistant nurses’, physicians’ and psychologists’ ratings regarding their interest in breastfeeding (*p* = 0.002) as well as the importance of their role in providing breastfeeding support (*p* < 0.001). Please see Table 2 for further information.

**Table 2:** Participants in median rate from 1—10 of interest in breastfeeding, how important they thought they were for better breastfeeding support, increased interest and if they got new tools

Profession	*n*	Variable	Median	Range
Midwife	142 142	Interest Important role	8 10	2—10 3—10
	139 140	Increased interest New tools	9 9	1—10 3—10
Registered nurse	7 7	Interest Important role	10 10	8—10 8—10
	7 7	Increased interest New tools	10 10	3—10 3—10
Specialist nurse	14	Interest	8.5	6—10
	14	Important role	9.5	6—10
	14	Increased interest	9	5—10
	14	New tools	8.5	7—10
Assistant nurse	60	Interest	9	2—10
	60	Important role	10	4—10
	58 58	Increased interest New tools	10 10	1—10 3—10
Physician	12	Interest	6.5	3—10
	12	Important role	6	4—9
	12	Increased interest	6.5	5—10
	12	New tools	7	5—9
Psychologist	2	Interest	6.5	6—7
	2	Important role	6.5	6—7
	2	Increased interest	7.5	7—8
	2	New tools	7.0	7—7

A positive correlation was seen between the HCPs interest in breastfeeding and whether they considered their role in improving breastfeeding support to be important (r_s_ = 0,579, *p* < 0,001). After the training program, the HCPs estimated that their interest in breastfeeding had increased according to a median (range) of 9 (1–10) and considered that they had received new tools for breastfeeding support according to a median of 9 (3–10) points. The Kruskal-Wallis test showed a significant difference between the HCPs’ ratings regarding increased interest in breastfeeding (*p* = 0.031) and if they experienced they had received new tools for breastfeeding support (*p* = 0.005). Please see Table 2 for further information.

A positive correlation was seen between HCPs interest in breastfeeding before the training and whether they felt that their interest in breastfeeding had increased due to the training program (r_s_ = 0.445, *p* < 0.001), and between their interest and whether they felt that they had been given new tools by the training to provide better breastfeeding support (r_s_ = 0.363, *p* < 0.001).

**Positive evaluation of the training program.** Categories on what was particularly good about the training program by different participating professions are presented in Table 3.

**Table 3:** Categories on what was particularly good about the training program by different participating professions (155 individuals)

Categories	Professions					
	Midwives *n* = 92 (%)	Reg. nurses *n* = 3 (%)	Spec. nurses *n* = 11 (%)	Assis. Nurses *n* = 39 (%)	Physicians *n* = 8 (%)	Psychologists *n* = 2 (%)
Group discussions	54 (59)	2 (67)	6 (55)	21 (54)	5 (63)	1 (50)
The structure of the training program	45 (49)	2 (67)	4 (36)	20 (51)	4 (50)	0
Inspiration to work with breastfeeding support	19 (21)	0	3 (27)	9 (23)	0	0
New knowledge about breastfeeding	16 (17)	0	5 (45)	12 (31)	4 (50)	1 (50)
Useful material	19 (21)	0	1 (9)	4 (10)	1 (13)	1 (50)
Mixed professions	20 (22)	1 (33)	8 (73)	13 (33)	3 (38)	0

### Group discussions

The HCPs regarded the group discussions of the case scenarios as both important and fruitful. These sessions created a space for the HCPs to exchange attitudes, perspectives, and experiences, fostering mutual learning and insight. The opportunity for structured reflection was valuable, enabling a deeper engagement with the content. Additionally, the interactive format promoted active participation and collegial dialogue, contributing to a more meaningful and effective learning experience. One midwife wrote:” To watch the videos first and then go through everything again. Everything was very interesting. It gave me new inspiration to continue supporting breastfeeding women.”

### The structure of the training program

The HCPs stated that the schematic structure of the training program was well designed and effective. It began with short films about breastfeeding, which participants had viewed in advance, allowing them to come prepared with a basic understanding of the subject. This was followed by a structured repetition of the film content during the training day, reinforcing core messages and facilitating knowledge retention. The program then progressed to group discussions of case scenarios, which encouraged sharing of diverse clinical experiences. Finally, the whole-class discussions provided an opportunity to synthesize insights from the smaller groups, clarify uncertainties and explore different approaches. A midwife stated: “It is good to repeatedly hear the same information.”

### Inspiration to work with breastfeeding support

The HCPs stated that the training program served as a source of inspiration for their continued work with breastfeeding support. It reinforced the importance of their role in promoting and supporting breastfeeding in clinical practice. Additionally, the participants noted that the training helped them recall knowledge and evidence-based practices that had previously been set aside or faded from everyday use. This reactivation of existing knowledge was seen as both valuable and necessary, contributing to a renewed sense of confidence and competence in their professional responsibilities One midwife wrote: “This training raises the status of breastfeeding. How important breastfeeding is!” Another midwife shared: The training program gave me more motivation to work with breastfeeding support.”

### New knowledge about breastfeeding

The HCPs stated that the training program day provided them with new knowledge about breastfeeding and breastfeeding support, enhancing their understanding of both practical and theoretical aspects. The participants emphasized that the content introduced updated information and evidence-based strategies that they had not previously encountered. The training day was described as a motivating experience that not only deepened their expertise but also strengthened their commitment to promoting breastfeeding and supporting mothers more effectively in their clinical work. One assistant nurse wrote: “I have gained new thoughts on what and how I can talk about breastfeeding with parents”.

### Useful material

Further, the HCPs highly valued the information material provided. They described it as clear, relevant, well-structured and offering practical guidance. The material was seen as a useful resource for reinforcing key messages about breastfeeding. The participants also expressed that the material would be beneficial to use as tools in their ongoing work with breastfeeding support. One assistant nurse wrote: It was good to see the films and later get a repetition of its content. Everything was very interesting!” and a midwife shared: “The flipchart is a fantastic tool”.

### Mixed professions

The HCPs stated that it was particularly valuable that individuals from different professional backgrounds participated in the training program. They emphasized that having a mix of roles—such as midwives, nurses, physicians, and other HCPs enriched the discussions and allowed for a broader exchange of perspectives. This interdisciplinary approach was seen as essential for fostering a shared understanding and promoting cohesive, team-based care. The participants expressed a clear need for a dedicated arena where they could come together across professional boundaries, strengthen interprofessional collaboration, and align their approaches to breastfeeding support. The training program was therefore not only an educational opportunity, but also a much-appreciated forum for team-building and mutual learning One physician wrote: “It was particularly good to gather with representatives from all parts of the care chain”. One assistant nurse wrote: “It was good to meet different professions and share experiences. It provides better collaboration between different professions.” One specialist nurse wrote: It was good to meet staff from other units and discuss how we work.”

### Suggestions on how to improve the training program

For information about categories on how to improve the training program by different participating professions, please see Table 4. The HCPs had less improvement suggestions to share than comments on what was particularly good about the training program. However, some HCPs gave comments on this question and their answers resulted in five categories.

**Table 4:** Categories on how to improve the training program by different participating professions (60 individuals)

Categories	Professions					
	Midwives *n* = 39	Reg. nurses *n* = 0	Spec. nurses *n* = 3	Assis. nurses *n* = 14	Physicians *n* = 3	Psychologists *n* = 1
Fewer cases, more time for discussions	10		1	3	1	
Lack of participants of different professions	8		1	1	1	
Practical exercises and tips	9			4	1	
New information	8			1		1
Breaks during the day	3		1	6		

### Fewer cases, more time for discussions

The HCPs stated that there were too many case scenarios and that they would have wanted more practical tips on breastfeeding support. One midwife wrote: “Shorten the cases. Three is enough, four is too many. It was a long day when you’re not used to sitting still and listen”.

### Lack of participants of different professions

The HCPs stated that it was valuable to meet up with other professions and they were dissatisfied with the fact that some professions, or units, were few or did not participate at all. One midwife wrote: “It would have been better if the midwives from the health care centers also joined” and another midwife stated that “there were too few physicians who participated”.

### Practical exercises and tips

The HCPs stated that they would have wanted more practical information on breastfeeding support and tips on what to do when breastfeeding problems occurs. One midwife wrote: “I would have wanted to know more about hand milking and other practical tips”. Another midwife wrote: “What to do when the woman is in pain…what to do when the baby does not latch at all and when the situation becomes increasingly chaotic.”

### New information

Also, the HCPs wished for further information that had not been presented or discussed during the day and expressed a need for more evidence-based facts about breastfeeding. One psychologist wrote: “I would have wanted more clear links to relevant and up-to-date research.”

### Breaks during the day

The HCPs stated that they would have wanted more breaks throughout the day. One physician put it as follows: “A little more leg stretching and a cookie for the afternoon coffee.”

### Suggestions for improvement regarding breastfeeding support at work

During the interprofessional group discussions, the groups submitted their suggestions for improvement regarding breastfeeding support at their unit and/or in the care chain. In the analysis of these suggestions, three categories were identified: *Material things that can be improved*, *Changes in the way HCPs work* and *Employee welfare*.

#### Material things that can be improved

The HCPs stated that breastfeeding information and information about skin-to-skin for parents in different formats were important. They suggested updated information, both online and as brochures, and that recurring information would be broadcasted on a television in both common areas and in the patient rooms. They also wished that the information should be available in several languages, apart from Swedish. Another improvement suggested by the HCPs was to remove sleeping pods for the babies and instead offer baby slings to the families to increase skin-to-skin care and to make it easier for parents to learn their baby’s cues. They also mentioned the physical environment and suggested improvements for a more home-like feeling such as the need for specific nursing chairs, possibility of dimmed illumination and larger beds to enable secure bed sharing. To improve the care within the care chain, the HCPs suggested standardized training material and information as well as check lists of information that must be given to parents before discharge.

#### Changes in the way the HCPs work

The HCPs were coherent about that breastfeeding and the nine innate steps of the infant should be prioritized above other chores. For example, they suggested prioritizing the first breastfeeding session after birth and that oftentimes tasks such as repairing vaginal tears can be postponed. They also stressed the possibility to improve the strive for zero-separation when either mother or baby are in need for more advanced healthcare, i.e. at the intensive care unit or the NICU. The HCPs also stressed the importance of adequate pain relief for both mothers and infants after cesarean section or vacuum extraction.

#### Employee welfare

For the HCPs, it was important that inter-professional training program dedicated for breastfeeding should be recurrent and that new staff should undergo the same training. Other things to be improved according to the HCPs was more staff or less patients/HCP at each unit/health clinic, which would leave more time for breastfeeding support. They also wished for clear memos on various care situations such as zero-separation and the phasing out of infant formula. The HCPs also stated that the collaboration between units as well as outpatient clinics should be improved, specifically between prenatal care and child health centers and that it was important to involve the partner during breastfeeding education or support.

## Discussion

This study describes how participating HCPs experienced a breastfeeding training program and their suggestions on how to improve breastfeeding support at work. After the training program, the HCPs estimated that their interest in breastfeeding had increased and they considered that they had received new tools for breastfeeding support, although there was a difference between different professions. The difference between the professions is probably due to that different HCPs have varying levels of education in breastfeeding and also work at different levels of proximity to the patients.

Education in breastfeeding support has been identified as significantly influencing mothers’ decisions to breastfeed [14]. While the BFHI has been implemented in many countries, numerous wards still need to enhance their efforts to support breastfeeding. A previous study showed that HCPs interest in breastfeeding was linked to their ability to provide effective breastfeeding support [15]. These circumstances underscore the importance of equipping HCPs with engaging knowledge and communication techniques [16]. Additionally, they highlight the need for inter-professional learning to dispel myths and misunderstandings surrounding breastfeeding, thereby providing holistic and supportive care [17]. The training program aimed to address all these aspects. The HCPs stated that they would have wanted more new information and evidence-based information. The fact that some HCPs wanted more evidence-based information showed that they may lack basic knowledge about breastfeeding and carry with them preconceived notions of what breastfeeding means. Some HCPs stated that they would have wanted more practical exercises and tips. This is something we, as midwives and lecturers, often hear during lessons on breastfeeding. There is a desire for quick solutions to problems or difficulties when providing breastfeeding support. Previous research has shown that quick fixes can lead to negative outcomes, as for example when HCPs uses their hands to induce a breastfeeding and connects the breast to the baby’s mouth [18] neglecting the baby’s innate reflexes, as described by Widstroem et al. [19]. Breastfeeding is a delicate interaction between mother and child that cannot be forced or rushed [20]. Instead, the program focuses on preventive efforts to promote breastfeeding. This includes protocols aligned with the Ten Steps to Successful Breastfeeding and providing skin-to-skin care for the newborn.

Older age, having children, and personal breastfeeding experiences seem to be better indicators of breastfeeding knowledge and attitudes than education [21]. However, it has been shown that it is important to reflect on your own experiences and prejudices in order to become a good breastfeeding supporter [22]. Such an exercise was included in the program, but may need to be expanded.

Indeed, the training program included group discussions about different cases and we also believe, just as the HCPs stated, that we included too many scenarios. In total, we presented 17 cases (Appendix 1) and it would be better to use 12 number of cases to give enough room for discussion.

Our goal was to provide uniform training to all staff in the care chain and invited all HCPs to join. The staff that was most difficult to get on board were the physicians and midwives working policlinically with antenatal care. This was also reflected in the participants’ desire to have more representatives from different professions participating in the training program. Nevertheless, our study showed that it is possible to mix HCPs from different professions.

It is always important to schedule for breaks when organizing a training program like in the present study. Some HCPs stated that there were not enough breaks and that it got tiresome. Worth mentioning, was also that nine HCPs specifically wrote that they would have wanted more *fika*. *Fika* is a Swedish tradition of taking joint breaks during the working day where you have coffee/tea and a sandwich or something sweet as a fruit, bun or cookie. In the afternoon of the training program, the HCPs were offered plain coffee/tea which might been the reason for some’s disappointment.

### Limitations

Although it is well known that breastfeeding has several benefits for both mother and child, the topic is not integrated in the education for physicians in Sweden. Hence it is also difficult to motivate physicians to partake in training sessions as the one described here, even though physicians often play a crucial role in how infants are fed. The main limitation of this study was that we could not motivate all HCPs to take part in the training program nor in the study, specifically not the physicians as only five out of plenty were present. To achieve enduring breastfeeding support improvements, it would be necessary to include all personnel in the care chain, since the hierarchical structure of healthcare is an obstacle to bottom-up change. Another limitation is that the present study was part of a larger project, *The Breastfeeding Study* [10], where data is processed continuously according to ability. The data, analysed for the present study, were collected between 2018 and 2019 and we cannot rule out the possibility that HCPs of today would have responded differently.

## Conclusion

The present breastfeeding training program was shown to be useful to all kinds of HCPs. The training program increased the participants interest in breastfeeding and provided them with tools in their continued work with breastfeeding support. Nevertheless, it is vital to free up time for the staff to participate in such a training program and to promote engagement for breastfeeding as an important topic for all HCPs working with women and children.

## Supplementary Information

Below is the link to the electronic supplementary material.

Supplementary Material 1

## Data Availability

The dataset used during the current study are available from the corresponding author on reasonable request.
