# Supplementary material for: Experiences of healthcare professionals in a breastfeeding training program
Source: Int Breastfeed J. 2025 Aug 12;20:61. doi: 10.1186/s13006-025-00760-2 (PMC12341093; doi:10.1186/s13006-025-00760-2)
Supplement: Supplementary file 1 — Supplementary Material 1 [file 13006_2025_760_MOESM1_ESM.docx]

**Information for parents**

Links to brochures:

<http://pdf-flip.se/SPBS_2018/8/>

<http://pdf-flip.se/SPS2/>

Link to video:

https://media.medfarm.uu.se/play/kanal/456/video/8734

**Education for health care professionals part one: web lectures**

Table 1. The content of the web lectures.

|  | Basic web lectures (10 minutes each) |
| --- | --- |
| 1 | Breastfeeding and professional support |
| 2 | Breastfeeding and health |
| 3 | Breastfeeding and self-efficacy |
| 4 | The very first breastfeeding session |
| 5 | Breastfeeding, skin-to-skin contact, and kangaroo mother care |
| 6 | Attachment, skin-to-skin contact, and breastfeeding |
| 7 | Expressing milk by hand, pumping, and supplementation |
| 8 | How to prevent breastfeeding problems |

Table 2. The content of additional web lectures tailored for the child health clinics.

|  | Additional web lectures |
| --- | --- |
| 1 | Breastfeeding recommendations and the WHO code |
| 2 | Breastfeeding and complementary feeding |
| 3 | Breastfeeding patterns |
| 4 | Breastfeeding and growth |
| 5 | Breastfeeding, sleep, and infant crying |

Link to the video channel with web lectures:

<https://media.medfarm.uu.se/play/kanal/414/video/7511>

**Education for health care professionals part two: a summary of the program for the training day in breastfeeding and skin-to-skin contact**

8 am: Introduction; summary of web lectures; key points

9 am: In your small group of 3–5 persons, work on the tasks below:

*A. Personal experience of breastfeeding:* What experiences and attitudes do you have toward breastfeeding? Were you breastfed yourself? Have you breastfed? What experiences do you of have of breastfeeding among relatives, friends, and acquaintances?

*B. Breastfeeding and your professional role:* How is breastfeeding included in your professional role? What are the problems related to breastfeeding and skin-to-skin care at your unit? How could these problems be solved?

*C. What changes would you like to introduce at your workplace in order to make it easier for mothers to breastfeed?* Write down possible improvements that could be implemented within the existing resources.

10 am: Rejoin the large group to discuss and present the tasks from the small groups.

11 am: Lecture and discussion: What is breastfeeding support?

12 am: Lunch

1 pm: In your small groups perform the following tasks:

*A. Read the brochures and suggest improvements.*

*B. Test the flipchart on each other and suggest improvements.*

*C. Read your three assigned scenarios and discuss how you could give good support to the parents in the form of emotional, informative, instrumental, and practical support.*

2.30 pm: Rejoin the large group to discuss all the scenarios from the small groups.

2:45 pm: Review and summary

**Breastfeeding scenarios**

Several breastfeeding scenarios are described below. Each group will be assigned three cases to work with. Discuss in your small group how to provide good support to these parents in the form of emotional, informative, instrumental, or practical support.

*(Note: The bulleted italicized list is the course facilitator’s list of important aspects assessed by the expert group that needed to be addressed during the discussion, in case the group members did not bring them up on their own.)*

1. Sonya’s first child was born in week 32. Now the baby is 40 weeks and on a neonatal follow-up visit. Sonya thinks the baby wants to suckle all the time, and has started giving the baby a pacifier. “She doesn’t leave me alone,” she says. “As soon as I put her down, she starts crying. I have to give her formula for her to be satisfied.”

- *Normalize the infant’s behavior*
- *Breastfeeding patterns*
- *Feeding cues*
- *Taking turns with partner*
- *Positive mirroring*
- *Laid-back breastfeeding (rather than seeing breastfeeding as performance)*

1. Aya comes for a follow-up visit with her six-week-old infant. The infant had low blood glucose at birth and was fed with formula. Aya struggles to make breastfeeding work, but the baby is fed almost entirely on formula.

- *Reduce formula feeding according to a plan and always start with breastfeeding*
- *Skin-to-skin contact (SSC)*
- *Start over, let the infant go through the later of the nine phases*
- *Breastfeed on early feeding cues*
- *Reduce formula quickly if the baby sucks at the breast*
- *Get off the clock*
- *Do not give the mother burdensome health advice to increase milk production*

1. Anna and Mats are caring for their newborn baby at the ward, and say that they intend to share the feeding of the infant equally when they are discharged from the hospital. When you enter the room, the child is in its bed with clothes on.

- *Parents taking turns in providing SSC*
- *Bring up the child’s perspective*
- *Normal baby behavior*
- *Explain how breastfeeding works*
- *Involve the partner in other parts*
- *What is gender equality?*
- *How to get enough rest*
- *Nipple confusion*

1. Emelie is caring for her preterm infant at the NICU. You feel that it is difficult to connect with her, and notice that she lacks emotional expression. Emelie is a single parent and smokes 10 cigarettes/day. The infant is almost never in SSC.

- *Low self-efficacy can be mistaken for low motivation for breastfeeding*
- *Positive feedback*
- *Relieve guilt regarding smoking and possible depression*
- *Encourage breastfeeding*
- *Many risk factors for not breastfeeding, even more support*
- *Risk factors for SIDS*

1. Karin has sore nipples. She cries when the baby, who is full term, begins to suck. When you ask how much it hurts, she answers maximum pain (10 on a 10-point scale).

- *Big latch, how to learn*
- *Mother shaping the breast*
- *SSC*
- *Pain relief*
- *Breastfeeding observation*
- *Follow-up, where?*

1. Adam was born in week 34 and is small for his gestational age. His mother pumps and feeds him with a cup. When she tries to breastfeed him, he screams and arches his body like a bow; he does not want to suck at the breast at all.

- *Remove the bottles from the unit?*
- *Start from the beginning with the nine phases and SSC*
- *Try in the morning*
- *Continue breastfeeding if he takes the breast, do not limit the suckling time*
- *Use a cup for extra milk*
- *SSC*
- *Laid-back breastfeeding*
- *Large amounts of milk that need to be reduced?*
- *Milk expression by hand before latch to soften the areola*

1. Sofia breastfeeds and says that the infant sucks well. The infant receives 23 mL every two hours with a tube. Sofia asks you if she should reduce the amount of milk.

- *Avoid exact amounts of milk*
- *Yes, if the infant sucks, reduce the milk amount*
- *How do you know the infant is sucking effectively?*
- *Breastfeeding observation/problems with observation*
- *How do you know the infant is getting milk?*
- *Test-weighing/observing the infant’s suckling behavior*
- *How to reduce additional milk volumes?*

1. Martin has become a father and feels very worried. His son is full term, weighs 2.2 kg, and is breastfeeding a lot. He follows you out into the corridor. “How do you know that the milk is coming?” he asks.

- *Normalize infant behavior, positive mirroring of what works*
- *Inform about the infant’s small stomach*
- *The infant's suckling gets the milk running*
- *The infant starts to urinate and defecate because of milk*
- *Try to encourage him to be supportive*

1. Mary’s milk is flowing and her infant wants to eat all the time. Mary says that the infant sleeps well during and between breastfeeding, preferably in the M-position on Mary’s chest. They really snuggle up together in the dark room.

- *Do nothing, mirror the positive interaction between them*
- *Safe co-sleeping*

1. Elvira has had her second child, who is being cared for at the ward. Elvira is doubtful if she wants to breastfeed. Breastfeeding the former child did not go very well. That child lost more than 10% in weight and was fed with formula at the maternity ward. The infant needed large amounts and was fed with a bottle. Then the baby didn’t want to suck. When Elvira put the baby to her breast, it just screamed. The beginning of motherhood was not at all like Elvira had imagined and is coloring the way she feels about her new baby and about breastfeeding.

- *Does not have to be the same as before*
- *Knowledge of infant behavior*
- *Mother already knows a lot about breastfeeding*
- *Address concerns, guilt relief*
- *Plan for support, where to turn?*
- *Problems with bottles*

1. Gustav comes on a follow-up visit with his son, who is two months old. When the boy was born, he was on the average curve for weight, but he has now slipped down one standard deviation.

- *Normalize*
- *How do you know the child is getting milk?*
- *Mirror the positive*
- *Reduce the stigma*

1. You are working in the labour ward and taking care of a mother. In the patient record, it states: “Does not want to breastfeed!” How do you support this mother when the baby is born?

- *It is always the woman who decides; she never needs to explain*
- *Maybe she wants to talk about it, maybe not*
- *The mother needs information about formula feeding and proximity*
- *Some may want to provide colostrum*
- *Hobby breastfeeding?*
- *Pump and bottle feed?*

1. Livia is on the postnatal ward with her newborn son Lukas. She has previously been depressed and is still on medication. In the middle of the night, she rings the bell. Lukas is trying to find the nipple, but does not latch on, or is popping on and off the breast. Livia thinks that everything feels overwhelming. She wants you to take the baby out of the room.

- *Who is best at determining about breastfeeding and medication?*
- *Abstinence symptoms in the baby*
- *Normal popping on and off the breast*
- *Shaping the breast*
- *Laid-back breastfeeding*
- *Normalizing*
- *Encourage her to not make major decisions at night*

1. Aisha is full term and three days old, and has lost 11% of her birthweight. The mother says that her daughter is suckling a lot; she is actually breastfeeding all the time.

- *How do you know that the child is getting enough milk?*
- *Do not limit suckling time*
- *Look at the baby, not only the numbers*
- *Other factors for big loss (intrapartum i.v. fluids)*
- *Signs of mother’s milk coming in?*

1. Michelle’s son was born preterm and was transferred directly to the neonatal ward. Michelle has severe preeclampsia.

- *Pumping/hand expressing before delivery*
- *When to start and how often*
- *Can mother-infant separation be avoided?*
- *Father SSC 24/7*
- *Picture of the infant for the mother in the recovery room*

1. Halima’s delivery ended very unexpectedly and suddenly in an emergency caesarean section, and she was given general anesthesia during surgery. Her husband, Hassan, comes to the ward with their newborn daughter. Hassan’s mother (Halima’s mother-in-law) also comes along. Hassan is dressed in a shirt, tie, and suit, and the mother-in-law carries the baby swaddled in a towel.

- *Don’t let cultural sensitivity come in the way of informing Hassan about the benefits of SSC*
- *SSC with Hassan or mother-in-law*
- *Dangers of swaddling*
- *Do relatives want to give sugar water? Inform about colostrum and exclusive breastfeeding*
- *A full-term infant has an energy reserve of about 500g*
- *Small stomach*
- *SSC with mother without delay*
- *Drugs in milk have the same concentration as in the mother’s blood (no need to throw out the first milk when mother returns)*

1. Amanda and Jonas had their second child in gestation week 32, two days ago. When you enter the room, their three-year-old daughter is there too. It turns out that the daughter is still breastfeeding.

- *A normal breastfeeding duration for humans*
- *There will be colostrum anyway*
- *Let the newborn breastfeed first*
- *Copious milk will come in faster*
